# Synthesis and crystal structures of manganese(I) carbonyl complexes bearing ester-substituted α-di­imine ligands

**DOI:** 10.1107/S2056989020010750

**Published:** 2020-08-11

**Authors:** Takatoshi Kanno, Tsugiko Takase, Dai Oyama

**Affiliations:** a Graduate School of Science and Engineering, Fukushima University, 1 Kanayagawa, Fukushima 960-1296, Japan; b Department of Natural Sciences and Informatics, Fukushima University, 1, Kanayagawa, Fukushima 960-1296, Japan

**Keywords:** crystal structure, manganese(I) complex, bipyridyl ligand, biquinolyl ligand, ester substituent

## Abstract

The crystal structures of two manganese(I) complexes with ester-substituted bi­pyridine or bi­quinoline supporting ligands are reported, namely, *fac*-bromido­tricarbon­yl(diethyl 2,2′-bi­pyridine-4,4′-di­carboxyl­ate-κ^2^ *N*,*N*′)mangan­ese(I), [MnBr(C_16_H_16_N_2_O_4_)(CO)_3_], **I**, and *fac*-bromido­tricarbon­yl(diethyl 2,2′-bi­quinoline-4,4′-di­carboxyl­ate-κ^2^ *N*,*N*′)manganese(I), [MnBr(C_24_H_20_N_2_O_4_)(CO)_3_], **II**. In both complexes, the manganese(I) atom adopts a distorted octa­hedral coordination sphere defined by three carbonyl C atoms, a Br^−^ anion and two N atoms from the chelating α-di­imine ligand. Both complexes show *fac* configurations of the carbonyl ligands. In **I**, the complex mol­ecules are linked by C—H⋯Br hydrogen bonds and aromatic π–π contacts. In **II**, intra­molecular C—H⋯O hydrogen bonds are present as well as inter­molecular C—H⋯O and C—H⋯Br hydrogen bonds and π–π inter­actions.

## Abstract 2

The structural comparison of two Mn^I^ tricarbonyl complexes bearing ester-substituted bi­pyridine or bi­quinoline ligands is reported.

## Chemical context   

Similar to carbonyl complexes of precious metals, such as ruthenium and rhenium, those with less expensive manganese are attracting attention for their application in CO_2 _reduction catalysts (Bourrez *et al.*, 2011 ▸) and as CO-releasing mol­ecules (CORMs) under external stimuli (Chakraborty *et al.*, 2014*a* ▸). For example, CORMs using manganese(I) carbonyl complexes controllably release CO by photoirradiation (Motterlini *et al.*, 2002 ▸). Considering their application *in vivo*, photo-CORMs are expected to utilize light at lower energy. In general, extended π-conjugation systems in organic ligands lead to redshifts of charge-transfer (CT) transition bands of manganese(I) carbonyl complexes (Chakraborty *et al.*, 2014*b* ▸). Therefore, it is essential to investigate the relationship between mol­ecular structures including π-conjugation systems and photophysical properties.
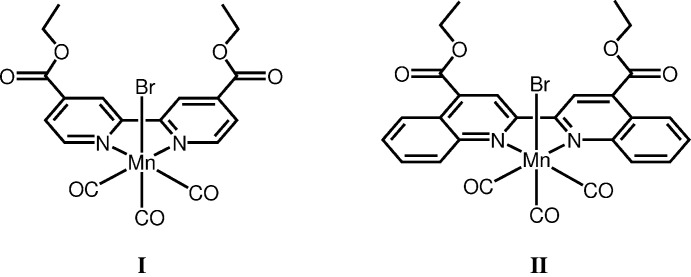


Thus, we focused on the comparison of bi­pyridines, which are prototypes of the α-di­imine ligand, and bi­quinolines with a more extended π-conjugation system. In addition, the introduction of ester groups into these ligands allows chemical adsorption with various metal oxides (Ardo & Meyer, 2009 ▸; Zhang *et al.*, 2006 ▸). In this study, we synthesized manganese(I) tricarbonyl complexes bearing two types of α-di­imine compounds, which contain both an ester substituent and different π-conjugation systems, *viz*. diethyl 2,2′-bi­pyridine-4,4′-dicarboxylate (debpy) and diethyl 2,2′-bi­quinoline-4,4′-di­dicarboxylate (debqn): *fac*-[MnBr(CO)_3_(debpy)] (**I**) and *fac*-[MnBr(CO)_3_(debqn)] (**II**). We successfully compared their crystal structures and photophysical properties. As expected, a CT band shift in the visible region was confirmed, depending on the size of the π-conjugation system in α-di­imine ligands. This finding will provide information in the future design of suitable complexes for a variety of photoreactions (Chakraborty *et al.*, 2014*b* ▸).

## Structural commentary   

**Figure 1:**
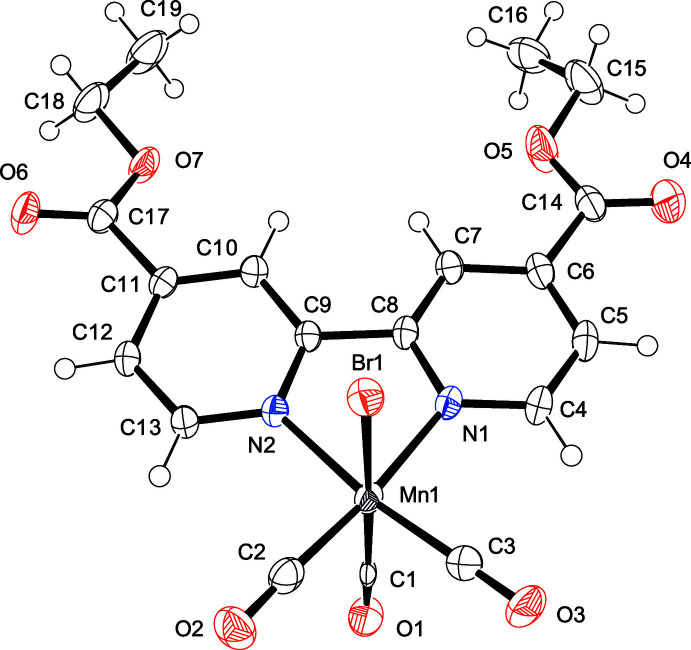
Mol­ecular structure of **I** with atom labeling and displacement ellipsoids drawn at the 50% probability level.

The mol­ecular structures of compounds **I** and **II** are shown in Figs. 1 ▸ and 2 ▸, respectively. In both complexes, the manganese(I) atoms exhibit distorted octa­hedral coordination geometries and display primary coordination spheres that are similar to those reported for other structurally related complexes (Chakraborty *et al.*, 2014*a* ▸; Walsh *et al.*, 2015 ▸). The metal–ligand bond lengths are similar to those previously reported for compounds of this type; in **I**, the Mn—N bond lengths are 2.046 (3) and 2.047 (2) Å, while in **II**, the Mn—N bond lengths are 2.063 (2) and 2.068 (2) Å. In **I** and **II**, the *fac* configuration of three CO ligands around the central manganese(I) atom is in agreement with their IR data. On the basis of their bond parameters, all CO ligands have typical triple-bond characters.

**Figure 3:**
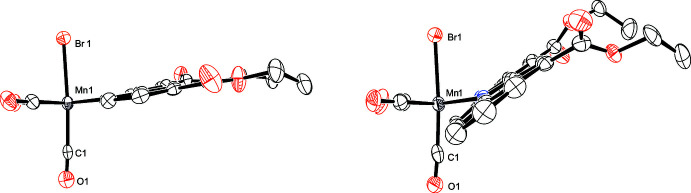
Side-on views of **I** (left) and **II** (right). H atoms are omitted for clarity.

The torsion angles between the equatorial plane and the debpy pyridyl ring in **I** (C3—Mn1—N1—C8 and C2—Mn1—N2—C9) are −169.17 (15) and 168.81 (14)°, respectively; the corresponding torsion angles in **II** (C3—Mn1—N1—C12 and C2—Mn1—N2—C13) are −147.52 (16) and 147.08 (17)°, respectively (Fig. 3 ▸). The large differences in torsion angles between **I** and **II** are mainly due to steric hindrance between H atoms (H1 and H10) in debqn, and the equatorial CO ligands (C3≡O3 and C2≡O2). On the basis of similar steric hindrance, comparable torsion angles [150.4 (15) and −150.7 (5)°] have been also observed in the related Re^I^ complex (Hallett *et al.*, 2011 ▸).

Despite similar mol­ecular skeletons, only **II** exhibits intra­molecular hydrogen bonds between the ester group and the quinolyl ring (Table 2 ▸). The C—C bond lengths of the coord­inated pyridyl rings in **I** [C6—C7 = 1.395 (3) Å and C10—C11 = 1.392 (3) Å] are considerably longer than the corresponding one in **II** [C10—C11 = 1.364 (4) Å and C14—C15 = 1.368 (4) Å]. This difference in structural parameters may eventually affect the intra­molecular hydrogen-bond formation.

## Supra­molecular features   

**Figure 4:**
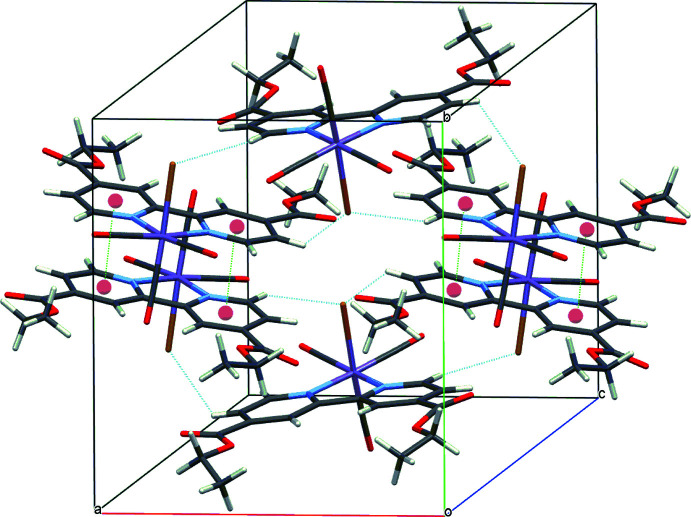
Crystal packing of **I** with C—H⋯Br hydrogen bonds (blue) and π–π contacts (green) shown as dashed lines; ring centroids are shown as red spheres.

In the crystal structure of **I**, complex mol­ecules are linked by pairs of weak C—H⋯Br hydrogen bonds (Table 1 ▸) and π–π inter­actions [*Cg*1⋯*Cg*2^iii^ = 3.683 (1) Å; *Cg*1 and *Cg*2 are the centroids of the N1/C4–C8 and N2/C9–C13 rings, respectively; symmetry code: (iii) 1 − *x*, −*y*, 1 − *z*], forming a three-dimensional supra­molecular structure (Fig. 4 ▸).

**Figure 5:**
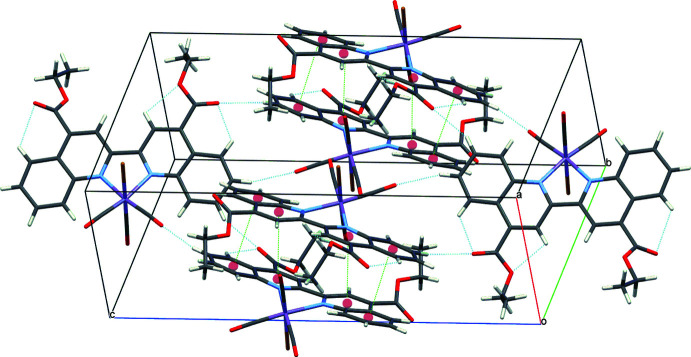
Crystal packing of **II** with C—H⋯Br hydrogen bonds (blue) and π–π contacts (green) shown as dashed lines; ring centroids are shown as red spheres.

In the crystal structure of **II**, there are weak C—H⋯O and C—H⋯Br hydrogen-bonding inter­actions (Table 2 ▸) as well as the above-mentioned intra­molecular hydrogen bonds. Additional π–π contacts are observed [*Cg*3⋯*Cg*4^iv^ = 3.732 (2) Å and *Cg*5⋯*Cg*6^iv^ = 4.002 (2) Å; *Cg*3, *Cg*4, *Cg*5 and *Cg*6 are the centroids of the C4–C9, C16–C21, N1/C8–C12 and N2/C13–C17 rings, respectively; symmetry code: (iv) 1 − *x*, 1 − *y*, −*z*]. These inter­actions lead to the formation of a three-dimensional network structure (Fig. 5 ▸).

## Database survey   

With respect to manganese(I) complexes with a bidentate bi­pyridine derivative ligand (N-N) of the form *fac*-[MnBr(CO)_3_(N-N)], some structures have been reported (CSD refcode POKGAZ; Chakraborty *et al.*, 2014*a* ▸, FUMKOQ and FUMKUW; Henke *et al.*, 2020 ▸, NIBSOJ; Lense *et al.*, 2018 ▸, XUVMUY and XUVNAF; Walsh *et al.*, 2015 ▸). However, no structures of bidentate bi­quinoline derivative–coordinated manganese(I) complexes have been reported; two structures of the corresponding rhenium(I) complexes have been determined by Hallett *et al.*, 2011 ▸ (EBANEC) and Kurz *et al.*, 2006 ▸ (XELXOC).

## Synthesis and crystallization   

The ligands, debpy and debqn, were prepared as described by Chandrasekharam *et al.* (2011 ▸) and Hoertz *et al.* (2006 ▸). The ligands were confirmed to be spectroscopically pure (by IR and ^1^H NMR analyses).

**Synthesis of I and II:** Compounds **I** and **II** were handled and stored in the dark to minimize exposure to light. For the synthesis of **I**, [MnBr(CO)_5_] (31 mg, 0.11 mmol) and debpy (33 mg, 0.11 mmol) were dissolved in CHCl_3_ (10 ml). The reaction mixture was stirred at 313 K for 14 h under N_2_. After the solvent was evaporated under reduced pressure, an excess of Et_2_O (30 ml) was added to the solution; then, the solution was allowed to stand at 253 K overnight. The resultant precipitate was collected by filtration, washed with Et_2_O, and then dried under vacuum (37 mg yield, 64%). Red crystals, suitable for the X-ray diffraction experiment, were grown by diffusion of *n*-hexane into an acetone solution of **I** for one week. FTIR (KBr pellet): νCO /cm^−1^ = 2028, 1918 (*br*) (C≡O), 1730 (C=O). UV–vis (CHCl_3_): λ /nm (∊ /M^−1^ cm^−1^) = 483 (3700), 367 (4100), 318 (21000), 247 (24000).

A similar reaction between [MnBr(CO)_5_] (8 mg, 0.029 mmol) and debqn (10 mg, 0.026 mmol) for 20 h afforded **II** (11 mg yield, 66%). Purple crystals, suitable for the X-ray diffraction experiment, were grown by diffusion of *n*-hexane into an acetone solution of **II** for one week. FTIR (KBr pellet): νCO /cm^−1^ = 2016, 1942, 1926 (C≡O), 1725 (C=O). UV–vis (CHCl_3_): λ /nm (∊ /M^−1^ cm^−1^) = 548 (3200), 383 (19000), 276 (37000).

## Refinement   

Crystal data, data collection and structure refinement details are summarized in Table 3 ▸. All hydrogen atoms were placed at calculated positions (C—H = 0.95—0.99 Å) and refined using a riding model with *U*_iso_(H) = 1.2*U*_eq_(C).

## Supplementary Material

Crystal structure: contains datablock(s) global, I, II. DOI: 10.1107/S2056989020010750/dj2012sup1.cif

Structure factors: contains datablock(s) I. DOI: 10.1107/S2056989020010750/dj2012Isup2.hkl

Supporting information file. DOI: 10.1107/S2056989020010750/dj2012Isup4.mol

Structure factors: contains datablock(s) II. DOI: 10.1107/S2056989020010750/dj2012IIsup3.hkl

Supporting information file. DOI: 10.1107/S2056989020010750/dj2012IIsup5.mol

CCDC references: 2021226, 2021225

Additional supporting information:  crystallographic information; 3D view; checkCIF report

## Figures and Tables

**Figure 2:**
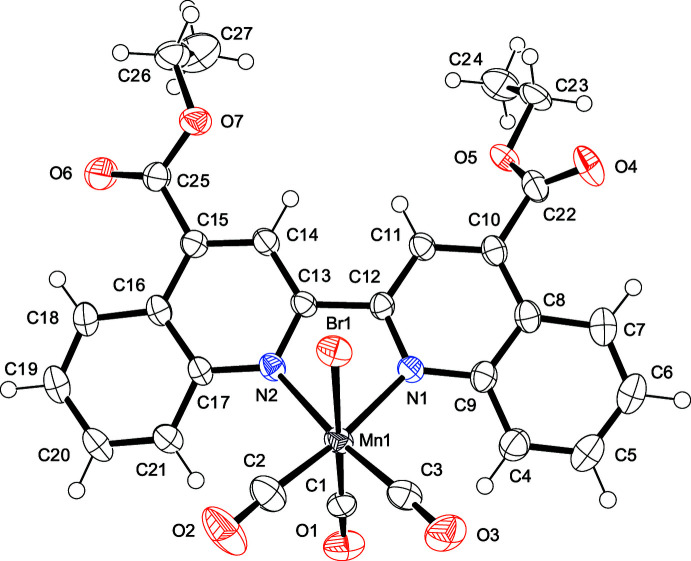
Mol­ecular structure of **II** with atom labeling and displacement ellipsoids drawn at the 50% probability level.

**Table 1:** Hydrogen-bond geometry (Å, °) for **I** [Chem scheme1]

*D*—H⋯*A*	*D*—H	H⋯*A*	*D*⋯*A*	*D*—H⋯*A*
C5—H2⋯Br1^i^	0.95	2.90	3.502 (3)	122
C13—H6⋯Br1^ii^	0.95	2.78	3.537 (3)	138

Symmetry codes: (i) 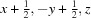; (ii) 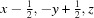.

**Table 2:** Hydrogen-bond geometry (Å, °) for **II** [Chem scheme1]

*D*—H⋯*A*	*D*—H	H⋯*A*	*D*⋯*A*	*D*—H⋯*A*
C7—H4⋯O4	0.95	2.44	3.040 (4)	121
C11—H5⋯Br1^i^	0.95	2.92	3.789 (3)	153
C14—H6⋯O7	0.95	2.33	2.659 (3)	100
C18—H7⋯O6	0.95	2.25	2.883 (5)	124
C19—H8⋯O2^ii^	0.95	2.47	3.373 (4)	160
C20—H9⋯O6^iii^	0.95	2.51	3.383 (4)	153

Symmetry codes: (i) 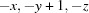; (ii) 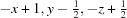; (iii) 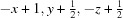.

**Table 3:** Experimental details

	**I**	**II**
Crystal data		
Chemical formula	[MnBr(C_16_H_16_N_2_O_4_)(CO)_3_]	[MnBr(C_24_H_20_N_2_O_4_)(CO)_3_]
*M*_r_	519.19	619.31
Crystal system, space group	Monoclinic, *P*2_1_/*a*	Monoclinic, *P*2_1_/*c*
Temperature (K)	93	93
*a*, *b*, *c* (Å)	11.7054 (7), 13.9151 (7), 13.3273 (8)	8.8953 (9), 12.0086 (13), 23.790 (3)
β (°)	110.347 (2)	95.794 (2)
*V* (Å^3^)	2035.3 (2)	2528.3 (5)
*Z*	4	4
Radiation type	Mo *K*α	Mo *K*α
μ (mm^−1^)	2.66	2.16
Crystal size (mm)	0.25 × 0.20 × 0.05	0.20 × 0.08 × 0.05

Data collection		
Diffractometer	Rigaku Saturn724	Rigaku Saturn70
Absorption correction	Multi-scan (*REQAB*; Rigaku, 1998 ▸)	Multi-scan (*REQAB*; Rigaku, 1998 ▸)
*T*_min_, *T*_max_	0.730, 0.875	0.461, 0.898
No. of measured, independent and observed [*F* ^2^ > 2.0σ(*F* ^2^)] reflections	20542, 4653, 4050	25401, 5757, 4100
*R*_int_	0.029	0.080
(sin θ/λ)_max_ (Å^−1^)	0.650	0.649

Refinement		
*R*[*F* ^2^ > 2σ(*F* ^2^)], *wR*(*F* ^2^), *S*	0.037, 0.098, 1.07	0.046, 0.134, 1.05
No. of reflections	4653	5757
No. of parameters	273	345
H-atom treatment	H-atom parameters constrained	H-atom parameters constrained
Δρ_max_, Δρ_min_ (e Å^−3^)	1.05, −0.56	0.79, −1.07

Computer programs: *CrystalClear* (Rigaku, 2015 ▸), *PROCESS-AUTO* (Rigaku, 1998 ▸), *SIR97* (Altomare *et al.*, 1999 ▸), *SHELXT2018/3* (Sheldrick, 2015*a* ▸), *SHELXL2018/3* (Sheldrick, 2015*b* ▸), *Mercury* (Macrae *et al.*, 2020 ▸), *ORTEP-3 for Windows* (Farrugia, 2012 ▸), *CrystalStructure* (Rigaku, 2019 ▸), *PLATON* (Spek, 2020 ▸) and *publCIF* (Westrip, 2010 ▸)

## supplementary crystallographic information

### fac-Bromidotricarbonyl(diethyl 2,2'-bipyridine-4,4'-dicarboxylate-κ2N,N')manganese(I) (I) Crystal data

**Table:**

[MnBr(C_16_H_16_N_2_O_4_)(CO)_3_]	*F*(000) = 1040.00
*M**_r_* = 519.19	*D*_x_ = 1.694 Mg m^−^^3^
Monoclinic, *P*2_1_/*a*	Mo *K*α radiation, λ = 0.71075 Å
*a* = 11.7054 (7) Å	Cell parameters from 5049 reflections
*b* = 13.9151 (7) Å	θ = 3.2–27.5°
*c* = 13.3273 (8) Å	µ = 2.66 mm^−^^1^
β = 110.347 (2)°	*T* = 93 K
*V* = 2035.3 (2) Å^3^	Block, red
*Z* = 4	0.25 × 0.20 × 0.05 mm

### fac-Bromidotricarbonyl(diethyl 2,2'-bipyridine-4,4'-dicarboxylate-κ2N,N')manganese(I) (I) Data collection

**Table:**

Rigaku Saturn724 diffractometer	4050 reflections with *F*^2^ > 2.0σ(*F*^2^)
Detector resolution: 28.626 pixels mm^-1^	*R*_int_ = 0.029
ω scans	θ_max_ = 27.5°, θ_min_ = 3.2°
Absorption correction: multi-scan (*REQAB*; Rigaku, 1998)	*h* = −15→15
*T*_min_ = 0.730, *T*_max_ = 0.875	*k* = −18→18
20542 measured reflections	*l* = −16→17
4653 independent reflections	

### fac-Bromidotricarbonyl(diethyl 2,2'-bipyridine-4,4'-dicarboxylate-κ2N,N')manganese(I) (I) Refinement

**Table:**

Refinement on *F*^2^	Secondary atom site location: difference Fourier map
*R*[*F*^2^ > 2σ(*F*^2^)] = 0.037	Hydrogen site location: inferred from neighbouring sites
*wR*(*F*^2^) = 0.098	H-atom parameters constrained
*S* = 1.07	*w* = 1/[σ^2^(*F*_o_^2^) + (0.055*P*)^2^ + 1.4779*P*] where *P* = (*F*_o_^2^ + 2*F*_c_^2^)/3
4653 reflections	(Δ/σ)_max_ = 0.001
273 parameters	Δρ_max_ = 1.05 e Å^−^^3^
0 restraints	Δρ_min_ = −0.56 e Å^−^^3^
Primary atom site location: structure-invariant direct methods	

### fac-Bromidotricarbonyl(diethyl 2,2'-bipyridine-4,4'-dicarboxylate-κ2N,N')manganese(I) (I) Special details

**Table:**

Geometry. All esds (except the esd in the dihedral angle between two l.s. planes) are estimated using the full covariance matrix. The cell esds are taken into account individually in the estimation of esds in distances, angles and torsion angles; correlations between esds in cell parameters are only used when they are defined by crystal symmetry. An approximate (isotropic) treatment of cell esds is used for estimating esds involving l.s. planes.
Refinement. Refinement was performed using all reflections. The weighted R-factor (wR) and goodness of fit (S) are based on F^2^. R-factor (gt) are based on F. The threshold expression of F^2^ > 2.0 sigma(F^2^) is used only for calculating R-factor (gt).

### fac-Bromidotricarbonyl(diethyl 2,2'-bipyridine-4,4'-dicarboxylate-κ2N,N')manganese(I) (I) Fractional atomic coordinates and isotropic or equivalent isotropic displacement parameters (Å^2^)

**Table:**

	*x*	*y*	*z*	*U*_iso_*/*U*_eq_	
Br1	0.61566 (2)	0.30862 (2)	0.74453 (2)	0.02885 (10)	
Mn1	0.59428 (3)	0.13106 (3)	0.76285 (3)	0.02227 (11)	
O1	0.56476 (18)	−0.07302 (16)	0.80205 (17)	0.0373 (5)	
O2	0.4659 (2)	0.17993 (16)	0.91224 (17)	0.0383 (5)	
O3	0.82383 (19)	0.14080 (17)	0.94807 (17)	0.0445 (6)	
O4	0.9317 (2)	0.0758 (2)	0.4214 (2)	0.0611 (8)	
O5	0.74278 (19)	0.0819 (2)	0.30486 (17)	0.0491 (6)	
O6	0.06293 (17)	0.21138 (15)	0.31716 (16)	0.0348 (5)	
O7	0.18898 (17)	0.15004 (15)	0.23897 (15)	0.0337 (5)	
N1	0.67488 (18)	0.10977 (15)	0.65084 (17)	0.0225 (4)	
N2	0.44701 (18)	0.13714 (14)	0.62383 (16)	0.0204 (4)	
C1	0.57649 (19)	−0.00038 (19)	0.78541 (18)	0.0184 (5)	
C2	0.5130 (2)	0.1609 (2)	0.8528 (2)	0.0281 (6)	
C3	0.7357 (2)	0.1367 (2)	0.8760 (2)	0.0300 (6)	
C4	0.7938 (2)	0.09076 (19)	0.6709 (2)	0.0277 (6)	
H1	0.846290	0.085051	0.743314	0.033*	
C5	0.8427 (2)	0.07926 (19)	0.5918 (2)	0.0286 (6)	
H2	0.926942	0.065380	0.609537	0.034*	
C6	0.7678 (2)	0.08816 (17)	0.4859 (2)	0.0248 (5)	
C7	0.6442 (2)	0.10676 (17)	0.4631 (2)	0.0227 (5)	
H3	0.590464	0.112142	0.391116	0.027*	
C8	0.6010 (2)	0.11726 (17)	0.5469 (2)	0.0212 (5)	
C9	0.4718 (2)	0.13526 (16)	0.53200 (19)	0.0195 (5)	
C10	0.3818 (2)	0.14873 (17)	0.4324 (2)	0.0218 (5)	
H4	0.401900	0.149195	0.369186	0.026*	
C11	0.2618 (2)	0.16152 (17)	0.42677 (19)	0.0218 (5)	
C12	0.2358 (2)	0.16067 (18)	0.5208 (2)	0.0228 (5)	
H5	0.154399	0.167911	0.518946	0.027*	
C13	0.3306 (2)	0.14909 (17)	0.6174 (2)	0.0219 (5)	
H6	0.312656	0.149609	0.681603	0.026*	
C14	0.8236 (2)	0.08021 (19)	0.4011 (2)	0.0300 (6)	
C15	0.7888 (3)	0.0781 (3)	0.2161 (3)	0.0509 (9)	
H7	0.812429	0.143292	0.200741	0.061*	
H8	0.861458	0.036113	0.234873	0.061*	
C16	0.6927 (3)	0.0403 (3)	0.1227 (3)	0.0487 (8)	
H9	0.720196	0.040532	0.061186	0.058*	
H10	0.619799	0.080493	0.106964	0.058*	
H11	0.673358	−0.025646	0.137198	0.058*	
C17	0.1594 (2)	0.17805 (19)	0.3228 (2)	0.0256 (5)	
C18	0.0934 (3)	0.1635 (3)	0.1343 (2)	0.0437 (8)	
H12	0.067516	0.231576	0.124367	0.052*	
H13	0.021624	0.123338	0.128791	0.052*	
C19	0.1454 (4)	0.1343 (3)	0.0510 (2)	0.0577 (11)	
H14	0.170249	0.066677	0.061456	0.069*	
H15	0.216324	0.174389	0.057323	0.069*	
H16	0.083590	0.142579	−0.020197	0.069*	

### fac-Bromidotricarbonyl(diethyl 2,2'-bipyridine-4,4'-dicarboxylate-κ2N,N')manganese(I) (I) Atomic displacement parameters (Å^2^)

**Table:**

	*U*^11^	*U*^22^	*U*^33^	*U*^12^	*U*^13^	*U*^23^
Br1	0.02690 (15)	0.02926 (15)	0.03069 (16)	−0.00070 (10)	0.01041 (11)	−0.00024 (10)
Mn1	0.01673 (19)	0.0283 (2)	0.01915 (19)	0.00051 (14)	0.00286 (14)	−0.00025 (14)
O1	0.0297 (11)	0.0444 (13)	0.0364 (11)	0.0040 (9)	0.0096 (9)	0.0013 (9)
O2	0.0380 (12)	0.0490 (13)	0.0330 (11)	−0.0006 (9)	0.0187 (10)	−0.0064 (9)
O3	0.0273 (11)	0.0608 (15)	0.0333 (11)	−0.0066 (10)	−0.0047 (9)	0.0106 (10)
O4	0.0359 (13)	0.099 (2)	0.0583 (16)	0.0226 (13)	0.0288 (12)	0.0239 (15)
O5	0.0283 (11)	0.0907 (19)	0.0335 (11)	−0.0039 (11)	0.0173 (9)	−0.0114 (12)
O6	0.0197 (9)	0.0471 (12)	0.0318 (11)	0.0090 (8)	0.0015 (8)	−0.0025 (9)
O7	0.0248 (10)	0.0503 (12)	0.0200 (9)	0.0118 (8)	0.0003 (7)	0.0020 (8)
N1	0.0160 (9)	0.0241 (10)	0.0241 (10)	0.0007 (8)	0.0027 (8)	−0.0001 (8)
N2	0.0164 (9)	0.0221 (10)	0.0215 (10)	−0.0004 (7)	0.0052 (8)	−0.0011 (8)
C1	0.0063 (9)	0.0344 (14)	0.0133 (10)	0.0023 (9)	0.0021 (8)	−0.0008 (9)
C2	0.0232 (13)	0.0321 (13)	0.0237 (13)	−0.0027 (10)	0.0015 (11)	0.0001 (10)
C3	0.0273 (13)	0.0344 (14)	0.0278 (13)	−0.0011 (11)	0.0089 (11)	0.0042 (11)
C4	0.0167 (11)	0.0316 (13)	0.0308 (13)	0.0030 (10)	0.0033 (10)	−0.0014 (11)
C5	0.0164 (11)	0.0268 (13)	0.0410 (15)	0.0020 (9)	0.0080 (11)	−0.0020 (11)
C6	0.0214 (12)	0.0197 (12)	0.0361 (14)	−0.0015 (9)	0.0136 (11)	−0.0030 (10)
C7	0.0192 (11)	0.0231 (12)	0.0258 (12)	−0.0022 (9)	0.0076 (10)	−0.0033 (9)
C8	0.0153 (11)	0.0210 (11)	0.0257 (12)	−0.0008 (9)	0.0051 (9)	−0.0019 (9)
C9	0.0160 (11)	0.0198 (11)	0.0223 (11)	−0.0012 (8)	0.0060 (9)	−0.0014 (9)
C10	0.0195 (11)	0.0227 (12)	0.0232 (12)	0.0013 (9)	0.0074 (10)	−0.0013 (9)
C11	0.0185 (11)	0.0217 (11)	0.0217 (12)	0.0006 (9)	0.0026 (9)	−0.0008 (9)
C12	0.0151 (11)	0.0246 (12)	0.0275 (13)	−0.0004 (9)	0.0058 (10)	−0.0018 (10)
C13	0.0177 (11)	0.0245 (12)	0.0238 (12)	0.0000 (9)	0.0078 (9)	−0.0011 (9)
C14	0.0264 (14)	0.0261 (13)	0.0433 (16)	0.0003 (10)	0.0194 (12)	0.0022 (11)
C15	0.0442 (19)	0.076 (3)	0.0455 (19)	−0.0077 (17)	0.0318 (16)	−0.0074 (17)
C16	0.054 (2)	0.065 (2)	0.0344 (17)	−0.0040 (17)	0.0246 (15)	0.0039 (16)
C17	0.0202 (12)	0.0275 (13)	0.0252 (13)	0.0000 (10)	0.0028 (10)	−0.0001 (10)
C18	0.0335 (16)	0.066 (2)	0.0220 (14)	0.0164 (15)	−0.0026 (12)	0.0040 (14)
C19	0.056 (2)	0.084 (3)	0.0250 (15)	0.034 (2)	0.0043 (15)	0.0070 (16)

### fac-Bromidotricarbonyl(diethyl 2,2'-bipyridine-4,4'-dicarboxylate-κ2N,N')manganese(I) (I) Geometric parameters (Å, º)

**Table:**

Br1—Mn1	2.5038 (5)	C6—C14	1.493 (4)
Mn1—C3	1.812 (3)	C7—C8	1.385 (4)
Mn1—C2	1.819 (3)	C7—H3	0.9500
Mn1—C1	1.877 (3)	C8—C9	1.477 (3)
Mn1—N1	2.046 (2)	C9—C10	1.391 (3)
Mn1—N2	2.047 (2)	C10—C11	1.392 (3)
O1—C1	1.054 (3)	C10—H4	0.9500
O2—C2	1.142 (3)	C11—C12	1.389 (4)
O3—C3	1.141 (3)	C11—C17	1.502 (3)
O4—C14	1.200 (3)	C12—C13	1.386 (3)
O5—C14	1.303 (4)	C12—H5	0.9500
O5—C15	1.461 (4)	C13—H6	0.9500
O6—C17	1.199 (3)	C15—C16	1.455 (5)
O7—C17	1.337 (3)	C15—H7	0.9900
O7—C18	1.466 (3)	C15—H8	0.9900
N1—C4	1.350 (3)	C16—H9	0.9800
N1—C8	1.358 (3)	C16—H10	0.9800
N2—C13	1.345 (3)	C16—H11	0.9800
N2—C9	1.353 (3)	C18—C19	1.495 (5)
C4—C5	1.373 (4)	C18—H12	0.9900
C4—H1	0.9500	C18—H13	0.9900
C5—C6	1.384 (4)	C19—H14	0.9800
C5—H2	0.9500	C19—H15	0.9800
C6—C7	1.395 (3)	C19—H16	0.9800
			
C3—Mn1—C2	88.72 (12)	C10—C9—C8	123.5 (2)
C3—Mn1—C1	91.66 (11)	C9—C10—C11	118.9 (2)
C2—Mn1—C1	90.24 (11)	C9—C10—H4	120.5
C3—Mn1—N1	95.38 (11)	C11—C10—H4	120.5
C2—Mn1—N1	173.53 (10)	C12—C11—C10	119.0 (2)
C1—Mn1—N1	94.63 (9)	C12—C11—C17	118.6 (2)
C3—Mn1—N2	171.65 (11)	C10—C11—C17	122.4 (2)
C2—Mn1—N2	96.79 (10)	C13—C12—C11	118.9 (2)
C1—Mn1—N2	94.58 (9)	C13—C12—H5	120.6
N1—Mn1—N2	78.60 (8)	C11—C12—H5	120.6
C3—Mn1—Br1	86.86 (9)	N2—C13—C12	122.7 (2)
C2—Mn1—Br1	86.10 (9)	N2—C13—H6	118.7
C1—Mn1—Br1	176.08 (7)	C12—C13—H6	118.7
N1—Mn1—Br1	89.12 (6)	O4—C14—O5	124.8 (3)
N2—Mn1—Br1	87.26 (6)	O4—C14—C6	122.6 (3)
C14—O5—C15	116.8 (2)	O5—C14—C6	112.6 (2)
C17—O7—C18	115.1 (2)	C16—C15—O5	108.2 (3)
C4—N1—C8	117.7 (2)	C16—C15—H7	110.1
C4—N1—Mn1	126.13 (17)	O5—C15—H7	110.1
C8—N1—Mn1	116.18 (16)	C16—C15—H8	110.1
C13—N2—C9	118.4 (2)	O5—C15—H8	110.1
C13—N2—Mn1	125.35 (17)	H7—C15—H8	108.4
C9—N2—Mn1	116.12 (15)	C15—C16—H9	109.5
O1—C1—Mn1	176.5 (2)	C15—C16—H10	109.5
O2—C2—Mn1	177.5 (2)	H9—C16—H10	109.5
O3—C3—Mn1	179.0 (3)	C15—C16—H11	109.5
N1—C4—C5	123.2 (2)	H9—C16—H11	109.5
N1—C4—H1	118.4	H10—C16—H11	109.5
C5—C4—H1	118.4	O6—C17—O7	124.9 (2)
C4—C5—C6	119.1 (2)	O6—C17—C11	123.3 (3)
C4—C5—H2	120.4	O7—C17—C11	111.7 (2)
C6—C5—H2	120.4	O7—C18—C19	107.4 (2)
C5—C6—C7	118.7 (2)	O7—C18—H12	110.2
C5—C6—C14	118.4 (2)	C19—C18—H12	110.2
C7—C6—C14	122.9 (2)	O7—C18—H13	110.2
C8—C7—C6	119.1 (2)	C19—C18—H13	110.2
C8—C7—H3	120.5	H12—C18—H13	108.5
C6—C7—H3	120.5	C18—C19—H14	109.5
N1—C8—C7	122.2 (2)	C18—C19—H15	109.5
N1—C8—C9	114.2 (2)	H14—C19—H15	109.5
C7—C8—C9	123.6 (2)	C18—C19—H16	109.5
N2—C9—C10	122.1 (2)	H14—C19—H16	109.5
N2—C9—C8	114.4 (2)	H15—C19—H16	109.5
			
C8—N1—C4—C5	0.3 (4)	C8—C9—C10—C11	177.5 (2)
Mn1—N1—C4—C5	−178.5 (2)	C9—C10—C11—C12	0.2 (4)
N1—C4—C5—C6	0.5 (4)	C9—C10—C11—C17	179.0 (2)
C4—C5—C6—C7	−1.2 (4)	C10—C11—C12—C13	1.3 (4)
C4—C5—C6—C14	177.1 (2)	C17—C11—C12—C13	−177.6 (2)
C5—C6—C7—C8	1.1 (4)	C9—N2—C13—C12	−0.8 (4)
C14—C6—C7—C8	−177.1 (2)	Mn1—N2—C13—C12	174.46 (18)
C4—N1—C8—C7	−0.5 (4)	C11—C12—C13—N2	−1.0 (4)
Mn1—N1—C8—C7	178.47 (18)	C15—O5—C14—O4	0.0 (5)
C4—N1—C8—C9	178.3 (2)	C15—O5—C14—C6	177.8 (3)
Mn1—N1—C8—C9	−2.8 (3)	C5—C6—C14—O4	−7.4 (4)
C6—C7—C8—N1	−0.2 (4)	C7—C6—C14—O4	170.8 (3)
C6—C7—C8—C9	−178.8 (2)	C5—C6—C14—O5	174.7 (2)
C13—N2—C9—C10	2.4 (3)	C7—C6—C14—O5	−7.1 (4)
Mn1—N2—C9—C10	−173.32 (18)	C14—O5—C15—C16	156.1 (3)
C13—N2—C9—C8	−177.2 (2)	C18—O7—C17—O6	0.5 (4)
Mn1—N2—C9—C8	7.0 (3)	C18—O7—C17—C11	179.7 (2)
N1—C8—C9—N2	−2.8 (3)	C12—C11—C17—O6	17.2 (4)
C7—C8—C9—N2	175.9 (2)	C10—C11—C17—O6	−161.6 (3)
N1—C8—C9—C10	177.6 (2)	C12—C11—C17—O7	−162.0 (2)
C7—C8—C9—C10	−3.7 (4)	C10—C11—C17—O7	19.2 (3)
N2—C9—C10—C11	−2.1 (4)	C17—O7—C18—C19	176.5 (3)

### fac-Bromidotricarbonyl(diethyl 2,2'-bipyridine-4,4'-dicarboxylate-κ2N,N')manganese(I) (I) Hydrogen-bond geometry (Å, º)

**Table:**

*D*—H···*A*	*D*—H	H···*A*	*D*···*A*	*D*—H···*A*
C5—H2···Br1^i^	0.95	2.90	3.502 (3)	122
C13—H6···Br1^ii^	0.95	2.78	3.537 (3)	138

Symmetry codes: (i) *x*+1/2, −*y*+1/2, *z*; (ii) *x*−1/2, −*y*+1/2, *z*.

### fac-Bromidotricarbonyl(diethyl 2,2'-biquinoline-4,4'-dicarboxylate-κ2N,N')manganese(I) (II) Crystal data

**Table:**

[MnBr(C_24_H_20_N_2_O_4_)(CO)_3_]	*F*(000) = 1248.00
*M**_r_* = 619.31	*D*_x_ = 1.627 Mg m^−^^3^
Monoclinic, *P*2_1_/*c*	Mo *K*α radiation, λ = 0.71075 Å
*a* = 8.8953 (9) Å	Cell parameters from 9084 reflections
*b* = 12.0086 (13) Å	θ = 3.0–27.7°
*c* = 23.790 (3) Å	µ = 2.16 mm^−^^1^
β = 95.794 (2)°	*T* = 93 K
*V* = 2528.3 (5) Å^3^	Block, purple
*Z* = 4	0.20 × 0.08 × 0.05 mm

### fac-Bromidotricarbonyl(diethyl 2,2'-biquinoline-4,4'-dicarboxylate-κ2N,N')manganese(I) (II) Data collection

**Table:**

Rigaku Saturn70 diffractometer	4100 reflections with *F*^2^ > 2.0σ(*F*^2^)
Detector resolution: 7.143 pixels mm^-1^	*R*_int_ = 0.080
ω scans	θ_max_ = 27.5°, θ_min_ = 3.0°
Absorption correction: multi-scan (*REQAB*; Rigaku, 1998)	*h* = −11→11
*T*_min_ = 0.461, *T*_max_ = 0.898	*k* = −15→15
25401 measured reflections	*l* = −30→30
5757 independent reflections	

### fac-Bromidotricarbonyl(diethyl 2,2'-biquinoline-4,4'-dicarboxylate-κ2N,N')manganese(I) (II) Refinement

**Table:**

Refinement on *F*^2^	Secondary atom site location: difference Fourier map
*R*[*F*^2^ > 2σ(*F*^2^)] = 0.046	Hydrogen site location: inferred from neighbouring sites
*wR*(*F*^2^) = 0.134	H-atom parameters constrained
*S* = 1.05	*w* = 1/[σ^2^(*F*_o_^2^) + (0.062*P*)^2^ + 0.6421*P*] where *P* = (*F*_o_^2^ + 2*F*_c_^2^)/3
5757 reflections	(Δ/σ)_max_ = 0.001
345 parameters	Δρ_max_ = 0.79 e Å^−^^3^
0 restraints	Δρ_min_ = −1.07 e Å^−^^3^
Primary atom site location: structure-invariant direct methods	

### fac-Bromidotricarbonyl(diethyl 2,2'-biquinoline-4,4'-dicarboxylate-κ2N,N')manganese(I) (II) Special details

**Table:**

Geometry. All esds (except the esd in the dihedral angle between two l.s. planes) are estimated using the full covariance matrix. The cell esds are taken into account individually in the estimation of esds in distances, angles and torsion angles; correlations between esds in cell parameters are only used when they are defined by crystal symmetry. An approximate (isotropic) treatment of cell esds is used for estimating esds involving l.s. planes.
Refinement. Refinement was performed using all reflections. The weighted R-factor (wR) and goodness of fit (S) are based on F^2^. R-factor (gt) are based on F. The threshold expression of F^2^ > 2.0 sigma(F^2^) is used only for calculating R-factor (gt).

### fac-Bromidotricarbonyl(diethyl 2,2'-biquinoline-4,4'-dicarboxylate-κ2N,N')manganese(I) (II) Fractional atomic coordinates and isotropic or equivalent isotropic displacement parameters (Å^2^)

**Table:**

	*x*	*y*	*z*	*U*_iso_*/*U*_eq_	
Br1	0.09534 (4)	0.75013 (2)	0.04399 (2)	0.03365 (12)	
Mn1	0.37395 (6)	0.74975 (3)	0.03376 (2)	0.02720 (14)	
O1	0.6977 (3)	0.77263 (19)	0.02409 (12)	0.0455 (6)	
O2	0.4042 (4)	0.8863 (2)	0.13644 (11)	0.0659 (8)	
O3	0.3198 (3)	0.96864 (19)	−0.02110 (11)	0.0478 (6)	
O4	−0.0202 (3)	0.4693 (2)	−0.19464 (10)	0.0522 (7)	
O5	0.1200 (2)	0.33393 (18)	−0.15029 (8)	0.0358 (5)	
O6	0.2414 (4)	0.2443 (2)	0.17911 (12)	0.0616 (9)	
O7	0.0767 (3)	0.25994 (16)	0.10327 (10)	0.0372 (5)	
N1	0.3222 (3)	0.64858 (19)	−0.03560 (9)	0.0255 (5)	
N2	0.3736 (3)	0.59349 (19)	0.07024 (9)	0.0265 (5)	
C1	0.5744 (4)	0.7601 (2)	0.02755 (14)	0.0331 (7)	
C2	0.3926 (4)	0.8304 (3)	0.09774 (14)	0.0400 (8)	
C3	0.3401 (4)	0.8825 (3)	−0.00119 (13)	0.0342 (7)	
C4	0.3949 (4)	0.7704 (3)	−0.10815 (15)	0.0380 (8)	
H1	0.459451	0.809647	−0.080705	0.046*	
C5	0.3815 (4)	0.8050 (3)	−0.16335 (15)	0.0468 (9)	
H2	0.437453	0.867719	−0.173866	0.056*	
C6	0.2868 (5)	0.7494 (3)	−0.20404 (16)	0.0489 (10)	
H3	0.273651	0.777152	−0.241603	0.059*	
C7	0.2124 (4)	0.6553 (3)	−0.19061 (13)	0.0417 (8)	
H4	0.151309	0.616168	−0.219148	0.050*	
C8	0.2262 (3)	0.6154 (3)	−0.13391 (12)	0.0308 (7)	
C9	0.3148 (3)	0.6779 (3)	−0.09197 (12)	0.0301 (6)	
C10	0.1627 (3)	0.5137 (2)	−0.11728 (11)	0.0283 (6)	
C11	0.1841 (3)	0.4822 (2)	−0.06195 (11)	0.0273 (6)	
H5	0.146315	0.412652	−0.050550	0.033*	
C12	0.2616 (3)	0.5524 (2)	−0.02203 (12)	0.0251 (6)	
C13	0.2867 (3)	0.5228 (2)	0.03829 (11)	0.0253 (6)	
C14	0.2265 (3)	0.4258 (2)	0.06018 (12)	0.0284 (6)	
H6	0.161956	0.379064	0.036264	0.034*	
C15	0.2605 (3)	0.3983 (2)	0.11581 (12)	0.0276 (6)	
C16	0.3641 (3)	0.4668 (2)	0.15020 (12)	0.0290 (6)	
C17	0.4186 (3)	0.5636 (2)	0.12506 (11)	0.0271 (6)	
C18	0.4134 (4)	0.4435 (3)	0.20761 (12)	0.0348 (7)	
H7	0.373534	0.381128	0.225729	0.042*	
C19	0.5173 (4)	0.5101 (3)	0.23671 (13)	0.0392 (8)	
H8	0.550979	0.492906	0.274893	0.047*	
C20	0.5755 (4)	0.6038 (3)	0.21124 (13)	0.0369 (8)	
H9	0.648519	0.649313	0.232167	0.044*	
C21	0.5280 (3)	0.6298 (3)	0.15676 (12)	0.0322 (7)	
H10	0.568787	0.693090	0.139770	0.039*	
C22	0.0748 (3)	0.4380 (3)	−0.15902 (12)	0.0342 (7)	
C23	0.0446 (5)	0.2485 (3)	−0.18631 (15)	0.0476 (10)	
H11	0.043775	0.269435	−0.226591	0.057*	
H12	−0.061144	0.238971	−0.177556	0.057*	
C24	0.1315 (5)	0.1432 (3)	−0.17463 (17)	0.0592 (11)	
H13	0.081839	0.082438	−0.196838	0.071*	
H14	0.135231	0.125364	−0.134315	0.071*	
H15	0.234523	0.152836	−0.185052	0.071*	
C25	0.1941 (4)	0.2929 (3)	0.13743 (13)	0.0324 (7)	
C26	0.0195 (4)	0.1492 (3)	0.11252 (15)	0.0412 (8)	
H16	−0.088192	0.144696	0.097417	0.049*	
H17	0.026769	0.133367	0.153545	0.049*	
C27	0.1078 (5)	0.0658 (3)	0.0841 (2)	0.0663 (12)	
H18	0.059641	−0.007362	0.085839	0.080*	
H19	0.210688	0.062260	0.103066	0.080*	
H20	0.111507	0.087199	0.044501	0.080*	

### fac-Bromidotricarbonyl(diethyl 2,2'-biquinoline-4,4'-dicarboxylate-κ2N,N')manganese(I) (II) Atomic displacement parameters (Å^2^)

**Table:**

	*U*^11^	*U*^22^	*U*^33^	*U*^12^	*U*^13^	*U*^23^
Br1	0.0304 (2)	0.03101 (19)	0.0392 (2)	0.00088 (13)	0.00171 (14)	−0.00383 (12)
Mn1	0.0290 (3)	0.0223 (2)	0.0293 (2)	−0.00015 (18)	−0.0018 (2)	−0.00277 (17)
O1	0.0346 (15)	0.0311 (13)	0.0702 (17)	0.0026 (10)	0.0027 (13)	0.0027 (11)
O2	0.085 (2)	0.0514 (16)	0.0556 (16)	0.0189 (15)	−0.0224 (15)	−0.0289 (13)
O3	0.0572 (16)	0.0252 (12)	0.0606 (15)	0.0045 (11)	0.0037 (13)	0.0056 (11)
O4	0.0465 (15)	0.0670 (18)	0.0383 (12)	0.0050 (13)	−0.0198 (11)	−0.0010 (12)
O5	0.0377 (12)	0.0377 (12)	0.0302 (11)	−0.0082 (10)	−0.0056 (9)	−0.0087 (9)
O6	0.070 (2)	0.0594 (18)	0.0497 (16)	−0.0249 (14)	−0.0218 (15)	0.0294 (12)
O7	0.0397 (14)	0.0282 (12)	0.0418 (13)	−0.0056 (10)	−0.0056 (11)	0.0058 (9)
N1	0.0261 (13)	0.0241 (12)	0.0259 (12)	0.0008 (10)	−0.0002 (10)	−0.0010 (9)
N2	0.0270 (13)	0.0259 (12)	0.0257 (12)	0.0024 (10)	−0.0014 (10)	−0.0011 (10)
C1	0.045 (2)	0.0181 (14)	0.0350 (16)	−0.0033 (13)	−0.0036 (15)	0.0017 (12)
C2	0.0393 (19)	0.0360 (18)	0.0423 (18)	0.0075 (15)	−0.0079 (15)	−0.0064 (15)
C3	0.0329 (17)	0.0296 (16)	0.0394 (17)	−0.0005 (14)	0.0002 (14)	−0.0057 (13)
C4	0.042 (2)	0.0348 (18)	0.0369 (17)	0.0002 (14)	0.0045 (15)	0.0036 (13)
C5	0.055 (2)	0.043 (2)	0.044 (2)	−0.0002 (18)	0.0138 (18)	0.0119 (16)
C6	0.061 (3)	0.051 (2)	0.0350 (18)	0.0026 (19)	0.0100 (18)	0.0139 (16)
C7	0.047 (2)	0.050 (2)	0.0285 (16)	0.0056 (17)	0.0015 (15)	0.0047 (14)
C8	0.0326 (17)	0.0323 (16)	0.0268 (14)	0.0072 (13)	0.0000 (13)	0.0004 (12)
C9	0.0298 (16)	0.0298 (15)	0.0301 (15)	0.0060 (13)	0.0009 (12)	0.0057 (12)
C10	0.0251 (15)	0.0330 (16)	0.0260 (14)	0.0062 (13)	−0.0011 (12)	0.0001 (12)
C11	0.0275 (15)	0.0263 (14)	0.0272 (14)	0.0022 (12)	−0.0022 (12)	0.0001 (12)
C12	0.0259 (15)	0.0248 (14)	0.0240 (13)	0.0029 (12)	−0.0002 (11)	0.0022 (11)
C13	0.0272 (15)	0.0216 (14)	0.0260 (13)	0.0005 (12)	−0.0022 (11)	−0.0018 (11)
C14	0.0311 (16)	0.0259 (14)	0.0267 (14)	0.0003 (13)	−0.0037 (12)	−0.0010 (11)
C15	0.0271 (16)	0.0260 (14)	0.0291 (14)	0.0018 (12)	0.0002 (12)	0.0007 (11)
C16	0.0298 (16)	0.0322 (16)	0.0239 (13)	0.0048 (13)	−0.0027 (12)	−0.0008 (12)
C17	0.0296 (16)	0.0289 (15)	0.0218 (13)	0.0018 (13)	−0.0021 (12)	−0.0022 (11)
C18	0.0425 (19)	0.0378 (17)	0.0236 (14)	0.0040 (15)	0.0002 (13)	0.0015 (12)
C19	0.046 (2)	0.045 (2)	0.0246 (14)	0.0085 (17)	−0.0044 (14)	−0.0025 (14)
C20	0.0384 (18)	0.0401 (18)	0.0300 (16)	0.0040 (15)	−0.0079 (14)	−0.0103 (13)
C21	0.0315 (17)	0.0310 (16)	0.0327 (15)	0.0005 (13)	−0.0025 (13)	−0.0033 (13)
C22	0.0305 (17)	0.0468 (19)	0.0246 (14)	0.0010 (15)	−0.0010 (13)	−0.0032 (13)
C23	0.051 (2)	0.057 (2)	0.0325 (17)	−0.0223 (18)	−0.0034 (17)	−0.0177 (15)
C24	0.075 (3)	0.045 (2)	0.056 (2)	−0.016 (2)	0.000 (2)	−0.0206 (18)
C25	0.0337 (18)	0.0309 (15)	0.0318 (16)	−0.0003 (14)	−0.0003 (13)	0.0052 (13)
C26	0.045 (2)	0.0267 (16)	0.051 (2)	−0.0054 (15)	0.0005 (16)	0.0047 (14)
C27	0.080 (3)	0.0310 (19)	0.091 (3)	0.003 (2)	0.023 (3)	−0.001 (2)

### fac-Bromidotricarbonyl(diethyl 2,2'-biquinoline-4,4'-dicarboxylate-κ2N,N')manganese(I) (II) Geometric parameters (Å, º)

**Table:**

Br1—Mn1	2.5146 (6)	C10—C22	1.506 (4)
Mn1—C2	1.798 (3)	C11—C12	1.398 (4)
Mn1—C1	1.809 (4)	C11—H5	0.9500
Mn1—C3	1.809 (3)	C12—C13	1.473 (4)
Mn1—N1	2.063 (2)	C13—C14	1.404 (4)
Mn1—N2	2.068 (2)	C14—C15	1.368 (4)
O1—C1	1.118 (4)	C14—H6	0.9500
O2—C2	1.135 (4)	C15—C16	1.429 (4)
O3—C3	1.144 (4)	C15—C25	1.508 (4)
O4—C22	1.195 (4)	C16—C17	1.416 (4)
O5—C22	1.323 (4)	C16—C18	1.419 (4)
O5—C23	1.457 (4)	C17—C21	1.414 (4)
O6—C25	1.190 (4)	C18—C19	1.358 (4)
O7—C25	1.318 (4)	C18—H7	0.9500
O7—C26	1.449 (4)	C19—C20	1.402 (5)
N1—C12	1.328 (4)	C19—H8	0.9500
N1—C9	1.382 (4)	C20—C21	1.358 (4)
N2—C13	1.333 (3)	C20—H9	0.9500
N2—C17	1.373 (3)	C21—H10	0.9500
C4—C5	1.371 (5)	C23—C24	1.493 (5)
C4—C9	1.394 (4)	C23—H11	0.9900
C4—H1	0.9500	C23—H12	0.9900
C5—C6	1.389 (6)	C24—H13	0.9800
C5—H2	0.9500	C24—H14	0.9800
C6—C7	1.363 (5)	C24—H15	0.9800
C6—H3	0.9500	C26—C27	1.478 (5)
C7—C8	1.425 (4)	C26—H16	0.9900
C7—H4	0.9500	C26—H17	0.9900
C8—C10	1.418 (4)	C27—H18	0.9800
C8—C9	1.422 (4)	C27—H19	0.9800
C10—C11	1.364 (4)	C27—H20	0.9800
			
C2—Mn1—C1	91.40 (15)	C14—C13—C12	122.4 (2)
C2—Mn1—C3	84.91 (15)	C15—C14—C13	120.3 (3)
C1—Mn1—C3	91.23 (14)	C15—C14—H6	119.9
C2—Mn1—N1	171.30 (13)	C13—C14—H6	119.9
C1—Mn1—N1	96.74 (12)	C14—C15—C16	118.8 (3)
C3—Mn1—N1	97.94 (11)	C14—C15—C25	118.5 (3)
C2—Mn1—N2	97.89 (12)	C16—C15—C25	122.6 (3)
C1—Mn1—N2	98.04 (11)	C17—C16—C18	118.9 (3)
C3—Mn1—N2	170.22 (12)	C17—C16—C15	117.3 (2)
N1—Mn1—N2	77.97 (9)	C18—C16—C15	123.8 (3)
C2—Mn1—Br1	85.68 (11)	N2—C17—C21	118.6 (3)
C1—Mn1—Br1	175.86 (9)	N2—C17—C16	122.5 (3)
C3—Mn1—Br1	85.59 (10)	C21—C17—C16	118.9 (3)
N1—Mn1—Br1	86.34 (7)	C19—C18—C16	120.2 (3)
N2—Mn1—Br1	85.29 (7)	C19—C18—H7	119.9
C22—O5—C23	117.3 (3)	C16—C18—H7	119.9
C25—O7—C26	116.8 (2)	C18—C19—C20	121.0 (3)
C12—N1—C9	118.5 (2)	C18—C19—H8	119.5
C12—N1—Mn1	112.39 (18)	C20—C19—H8	119.5
C9—N1—Mn1	127.65 (19)	C21—C20—C19	120.2 (3)
C13—N2—C17	118.1 (2)	C21—C20—H9	119.9
C13—N2—Mn1	111.33 (18)	C19—C20—H9	119.9
C17—N2—Mn1	128.58 (19)	C20—C21—C17	120.7 (3)
O1—C1—Mn1	176.2 (3)	C20—C21—H10	119.6
O2—C2—Mn1	176.4 (3)	C17—C21—H10	119.6
O3—C3—Mn1	177.1 (3)	O4—C22—O5	126.2 (3)
C5—C4—C9	120.6 (3)	O4—C22—C10	124.1 (3)
C5—C4—H1	119.7	O5—C22—C10	109.7 (2)
C9—C4—H1	119.7	O5—C23—C24	106.7 (3)
C4—C5—C6	120.6 (3)	O5—C23—H11	110.4
C4—C5—H2	119.7	C24—C23—H11	110.4
C6—C5—H2	119.7	O5—C23—H12	110.4
C7—C6—C5	120.7 (3)	C24—C23—H12	110.4
C7—C6—H3	119.7	H11—C23—H12	108.6
C5—C6—H3	119.7	C23—C24—H13	109.5
C6—C7—C8	120.2 (3)	C23—C24—H14	109.5
C6—C7—H4	119.9	H13—C24—H14	109.5
C8—C7—H4	119.9	C23—C24—H15	109.5
C10—C8—C9	117.9 (3)	H13—C24—H15	109.5
C10—C8—C7	123.7 (3)	H14—C24—H15	109.5
C9—C8—C7	118.4 (3)	O6—C25—O7	123.9 (3)
N1—C9—C4	119.7 (3)	O6—C25—C15	125.3 (3)
N1—C9—C8	121.0 (3)	O7—C25—C15	110.8 (2)
C4—C9—C8	119.3 (3)	O7—C26—C27	110.0 (3)
C11—C10—C8	119.2 (3)	O7—C26—H16	109.7
C11—C10—C22	118.8 (3)	C27—C26—H16	109.7
C8—C10—C22	122.1 (3)	O7—C26—H17	109.7
C10—C11—C12	119.9 (3)	C27—C26—H17	109.7
C10—C11—H5	120.0	H16—C26—H17	108.2
C12—C11—H5	120.0	C26—C27—H18	109.5
N1—C12—C11	122.9 (3)	C26—C27—H19	109.5
N1—C12—C13	114.9 (2)	H18—C27—H19	109.5
C11—C12—C13	122.1 (3)	C26—C27—H20	109.5
N2—C13—C14	122.5 (3)	H18—C27—H20	109.5
N2—C13—C12	115.1 (2)	H19—C27—H20	109.5
			
C9—C4—C5—C6	0.6 (6)	C12—C13—C14—C15	176.0 (3)
C4—C5—C6—C7	−4.0 (6)	C13—C14—C15—C16	−3.2 (4)
C5—C6—C7—C8	2.6 (6)	C13—C14—C15—C25	−179.7 (3)
C6—C7—C8—C10	−174.7 (3)	C14—C15—C16—C17	3.7 (4)
C6—C7—C8—C9	2.0 (5)	C25—C15—C16—C17	180.0 (3)
C12—N1—C9—C4	−171.2 (3)	C14—C15—C16—C18	−176.8 (3)
Mn1—N1—C9—C4	23.8 (4)	C25—C15—C16—C18	−0.5 (5)
C12—N1—C9—C8	8.9 (4)	C13—N2—C17—C21	170.9 (3)
Mn1—N1—C9—C8	−156.1 (2)	Mn1—N2—C17—C21	−26.4 (4)
C5—C4—C9—N1	−175.8 (3)	C13—N2—C17—C16	−6.5 (4)
C5—C4—C9—C8	4.1 (5)	Mn1—N2—C17—C16	156.1 (2)
C10—C8—C9—N1	−8.5 (4)	C18—C16—C17—N2	−178.4 (3)
C7—C8—C9—N1	174.6 (3)	C15—C16—C17—N2	1.1 (4)
C10—C8—C9—C4	171.6 (3)	C18—C16—C17—C21	4.2 (4)
C7—C8—C9—C4	−5.3 (4)	C15—C16—C17—C21	−176.3 (3)
C9—C8—C10—C11	2.4 (4)	C17—C16—C18—C19	−3.5 (4)
C7—C8—C10—C11	179.1 (3)	C15—C16—C18—C19	177.0 (3)
C9—C8—C10—C22	−176.4 (3)	C16—C18—C19—C20	1.2 (5)
C7—C8—C10—C22	0.3 (5)	C18—C19—C20—C21	0.3 (5)
C8—C10—C11—C12	3.0 (4)	C19—C20—C21—C17	0.5 (5)
C22—C10—C11—C12	−178.2 (3)	N2—C17—C21—C20	179.7 (3)
C9—N1—C12—C11	−3.3 (4)	C16—C17—C21—C20	−2.8 (4)
Mn1—N1—C12—C11	163.9 (2)	C23—O5—C22—O4	−1.8 (5)
C9—N1—C12—C13	174.6 (2)	C23—O5—C22—C10	178.8 (3)
Mn1—N1—C12—C13	−18.2 (3)	C11—C10—C22—O4	135.9 (3)
C10—C11—C12—N1	−2.7 (4)	C8—C10—C22—O4	−45.3 (5)
C10—C11—C12—C13	179.5 (3)	C11—C10—C22—O5	−44.7 (4)
C17—N2—C13—C14	7.2 (4)	C8—C10—C22—O5	134.1 (3)
Mn1—N2—C13—C14	−158.3 (2)	C22—O5—C23—C24	171.0 (3)
C17—N2—C13—C12	−171.3 (2)	C26—O7—C25—O6	−10.5 (5)
Mn1—N2—C13—C12	23.2 (3)	C26—O7—C25—C15	168.4 (3)
N1—C12—C13—N2	−3.5 (4)	C14—C15—C25—O6	159.9 (4)
C11—C12—C13—N2	174.4 (3)	C16—C15—C25—O6	−16.4 (5)
N1—C12—C13—C14	178.0 (3)	C14—C15—C25—O7	−18.9 (4)
C11—C12—C13—C14	−4.1 (4)	C16—C15—C25—O7	164.7 (3)
N2—C13—C14—C15	−2.4 (4)	C25—O7—C26—C27	−83.7 (4)

### fac-Bromidotricarbonyl(diethyl 2,2'-biquinoline-4,4'-dicarboxylate-κ2N,N')manganese(I) (II) Hydrogen-bond geometry (Å, º)

**Table:**

*D*—H···*A*	*D*—H	H···*A*	*D*···*A*	*D*—H···*A*
C7—H4···O4	0.95	2.44	3.040 (4)	121
C11—H5···Br1^i^	0.95	2.92	3.789 (3)	153
C14—H6···O7	0.95	2.33	2.659 (3)	100
C18—H7···O6	0.95	2.25	2.883 (5)	124
C19—H8···O2^ii^	0.95	2.47	3.373 (4)	160
C20—H9···O6^iii^	0.95	2.51	3.383 (4)	153

Symmetry codes: (i) −*x*, −*y*+1, −*z*; (ii) −*x*+1, *y*−1/2, −*z*+1/2; (iii) −*x*+1, *y*+1/2, −*z*+1/2.
